# Effects of Faraday cup deterioration on Sr and Cr isotope analyses by thermal ionization mass spectrometry

**DOI:** 10.1039/d4ja00153b

**Published:** 2024-06-10

**Authors:** Jonas M. Schneider, Thorsten Kleine

**Affiliations:** a Max Planck Institute for Solar System Research Justus-von-Liebig-Weg 3 D-37077 Göttingen Germany schneiderj@mps.mpg.de +49 551 384979 308; b Institute for Planetology, University of Münster Wilhelm-Klemm-Straße 10 D-48149 Münster Germany

## Abstract

By comparing data from an extensive set of Sr and Cr isotope measurements performed on two different thermal ionization mass spectrometers (TIMS), using three sets of Faraday cups with different usage histories, we assess the effects of Faraday cup deterioration on high-precision isotope measurements by TIMS. We find that dynamic ^84^Sr/^86^Sr and ^87^Sr/^86^Sr measurements provide stable and reproducible results over the entire 56 months of this study, regardless of which set of Faraday cups is used. By contrast, static ^84^Sr/^86^Sr and ^87^Sr/^86^Sr measurements lead to deviant results, drifts over time, and in general exhibit larger scatter. For the most part, these differences can be attributed to changing Faraday cup efficiencies. For the instruments of this study we find that the center cup is most affected, consistent with this cup often receiving the highest ion beam intensities during measurements conducted in our laboratory. For Cr isotopes, we find that the correlation between mass fractionation-corrected ^53^Cr/^52^Cr and ^54^Cr/^52^Cr ratios observed for static measurements in several prior studies also reflects different Faraday cup efficiencies. Again, the changing efficiency of predominantly the center cup can account for the observed drift and correlation in ^53^Cr/^52^Cr and ^54^Cr/^52^Cr. Multi-static Cr isotope measurements reduce this drift, but still result in a residual correlation between the two ratios, suggesting this correlation in part also reflects unaccounted mass fractionation effects.

## Abstract 2

We use measurements performed on two thermal ionization mass spectrometers (TIMS), and using three sets of Faraday cups, to assess the effects of Faraday cup deterioration on high-precision Cr and Sr isotope measurements by TIMS.

## Graphical abstract

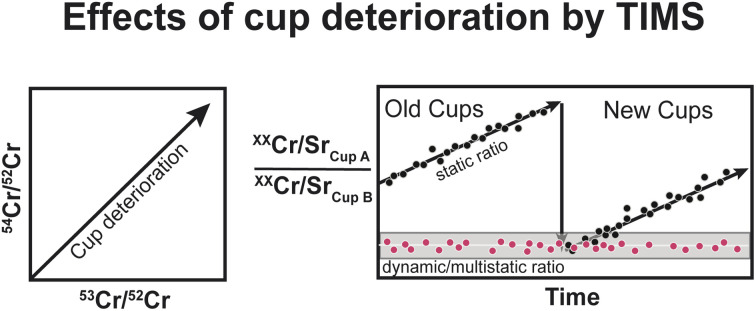

## 1. Introduction

Thermal ionization mass spectrometry (TIMS) is often the method of choice for high-precision isotope ratio measurements in geochemistry and cosmochemistry. This stems from the fact that the stability of the TIMS source allows for long-duration isotope analyses with small measurement uncertainties on individual isotope ratios. However, this in turn makes TIMS measurements susceptible to drifts in measured values that may be related to changes in instrument parameters during the course of an individual study. One such change is the deterioration of Faraday cups through the deposition of analyte atoms during measurements conducted with high ion beam intensities.^1^ Primarily due to different usage histories, the deterioration of individual cups likely varies over time, and so can result in a drift of measured isotope ratios on timescales of weeks to months, introducing systematic uncertainties in the final measured isotopic compositions of samples.^2,3^

Two elements that are frequently measured using TIMS are Sr and Cr. Strontium has four stable isotopes, namely ^84^Sr, ^86^Sr, ^87^Sr, and ^88^Sr with natural abundances of 0.56%, 9.86%, 7.00%, and 82.58%, respectively. Of these, ^87^Sr is produced by radioactive decay of ^87^Rb, making the ^87^Rb–^87^Sr useful for dating geological processes and as a geochemical tracer.^4^ In addition, the four Sr isotopes are produced by different stellar nucleosynthetic processes,^5^ and so measurements of ^84^Sr/^86^Sr are of interest to search for nucleosynthetic isotope anomalies in meteorites and meteorite components.^6–9^ Like Sr, Cr also has four stable isotopes, namely ^50^Cr, ^52^Cr, ^53^Cr, and ^54^Cr with natural abundances of 4.35%, 83.79%, 9.50% and 2.36%. Of these, ^53^Cr is produced by the decay of the short-lived, now-extinct radionuclide ^53^Mn (half-life ∼ 3.7 Ma), making the ^53^Mn–^53^Cr system an important chronometer for processes occurring in the first ∼20 Ma of the solar system.^10–13^ In addition, meteorites display widespread variations in ^54^Cr/^52^Cr, which are interpreted to predominantly be nucleosynthetic in origin.^14–16^ Together, this makes both Sr and Cr isotope measurements versatile tools to study a wide range of processes in geochemistry and cosmochemistry.

We routinely conduct Sr and Cr isotope measurements in our laboratory since 2018, using two different TIMS instruments and three sets of Faraday cups with different usage histories. Here we report an extensive set of Sr and Cr standard measurements acquired over this long period of time, including measurements with extensively used and new Faraday cups. By systematically investigating the effect of different cup settings using different sets of cups, this provides a unique opportunity to assess the significance and overall effect of cup deterioration on high-precision isotope measurements by TIMS.

## 2. Materials and methods

### 2.1. Materials and standards

The standards used throughout this study are the certified National Institute of Standards and Technology (NIST) standard reference materials (SRM) 3112a for Cr and 987 for Sr. Both standards were prepared as ∼500 ng μl^−1^ solutions using 6 M HCl; these solution were used for loading Sr and Cr onto the filaments. All measurements were performed using zone-refined Re filaments (99.999% Re, width 0.76 cm, purchased from H Cross Company, NJ 07074 USA), welded onto single filaments and cleaned by outgassing under vacuum (<1 × 10^−5^ mbar) at 4.5 A for 288 minutes using the Thermo Scientific filament degassing device.

### 2.2. Instrumental setup and measurement series

The isotope measurements of this study were conducted on two different instruments and using three sets of Faraday cups having different lifetimes. The first instrument is the Thermo Scientific Triton Plus at the University of Münster, for which two different sets of Faraday cups were used. The first set was used for ∼6 years for a variety of isotope measurements since installation of the instrument in 08/2015, including measurements of Ba, Nd, Sm, Cr, Sr, Dy, Er.^9,17–23^ As such, the measurements conducted with this instrument setup (hereafter ‘setup #1’) provide a good example of measurements utilizing cups with an intensive pre-history. In 12/2019, a new set of Faraday cups was installed on the Triton Plus in Münster (corresponding to ‘setup #2’), and new Sr and Cr isotope measurements were conducted starting immediately after the cup installation. The second instrument used in this study is a Thermo Scientific Triton XT installed at the Max Planck Institute for Solar System Research (MPS) in 09/2022. This instrument has been used for some additional ^87^Sr/^86^Sr measurements, which were performed immediately after installation of the instrument (‘setup #3’). Thus, comparing measurements using setup #1 with those using setups #2 and #3 allows for the direct assessment of potential effects of using Faraday cups of different ages and usage history on high-precision isotope measurements.

We report data from three measurement campaigns, which used the three setups described above. To this end, long-term ^87^Sr/^86^Sr measurements of SRM 987 are reported for all three setups, while ^84^Sr/^86^Sr as well as ^53^Cr/^52^Cr and ^54^Cr/^52^Cr measurements are reported for setups #1 and #2. In addition to these long-term measurements, we also performed two single sets of experiments where the ^84^Sr/^86^Sr and ^87^Sr/^86^Sr ratios were measured consecutively in static mode using five different magnetic settings with masses 84 through 88 collected in the center cup (see Table 1). For each magnet setting, 4–5 static measurements were conducted. The purpose of this experiment is to systematically assess how the use of specific Faraday cups affects the final measured isotope ratios and thereby test as to whether differences in deterioration among different Faraday cups can be identified. This experiment was performed using setups #1 and #2.

**Table 1:** Cup settings for Sr-isotopic measurements

Line	L4	L3	L2	L1	Center	H1	H2	H3	H4
^**87**^ **Sr/** ^**86**^ **Sr measurements**									
1				^84^Sr	^85^Rb	^86^Sr	^87^Sr	^88^Sr	
2			^84^Sr	^85^Rb	^86^Sr	^87^Sr	^88^Sr		
3		^84^Sr	^85^Rb	^86^Sr	^87^Sr	^88^Sr			

^**84**^ **Sr/** ^**86**^ **Sr measurements**									
1					^84^Sr	^85^Rb	^86^Sr	^87^Sr	^88^Sr
2			^84^Sr	^85^Rb	^86^Sr	^87^Sr	^88^Sr		

**Cup-efficiency experiment**									
84-center					^84^Sr	^85^Rb	^86^Sr	^87^Sr	^88^Sr
85-center				^84^Sr	^85^Rb	^86^Sr	^87^Sr	^88^Sr	
86-center			^84^Sr	^85^Rb	^86^Sr	^87^Sr	^88^Sr		
87-center		^84^Sr	^85^Rb	^86^Sr	^87^Sr	^88^Sr			
88-center	^84^Sr	^85^Rb	^86^Sr	^87^Sr	^88^Sr				

### 2.3. Strontium isotope measurements

The measurements of ^84^Sr/^86^Sr and ^87^Sr/^86^Sr ratios were performed independently of each other and using different cup configurations (see Table 1). A gain calibration was performed before each individual measurement session (corresponding to samples loaded onto a single sample wheel). For both measurements, 1 μl of the NIST SRM 987 solution containing ∼500 ng Sr was loaded onto each filament. The sample was dried at 1 A and topped with 1 μl of Ta_2_O_5_ activator solution consisting of 50 mg of 99.999% pure Ta_2_O_5_ powder suspended in 3 ml 0.5 M H_3_PO_4_. After the mixture dried, the filament was heated to glow for ∼1 s and is ready for measurement. For the ^87^Sr/^86^Sr measurements, the filaments were stepwise heated at 370 mA min^−1^ using the automatic heat function of the Triton until a signal of 0.2 V is reached on ^88^Sr. At this point the heating rate is reduced to 250 mA min^−1^ until a signal of 10 V on ^88^Sr is reached, and the measurement is started with a signal of 10.5 V on ^88^Sr. During the heating process, the lenses were focused and a peak center was performed at signals of 0.2 V and 10 V. For the measurement of ^84^Sr/^86^Sr, this procedure was the same, but the final signal was increased to 25–30 V on ^88^Sr and an additional lens focus was performed at a signal of 15 V. For both the ^84^Sr/^86^Sr and ^87^Sr/^86^Sr measurements, only Faraday cups connected to feedback resistors with 10^11^ Ω amplifiers were used, and no amplifier rotation was employed. Mass fractionation was corrected using the exponential law and ^86^Sr/^88^Sr = 0.1194.^24,25^

The ^84^Sr/^86^Sr measurements consisted of two lines of data acquisition, which provide two separate static ^84^Sr/^86^Sr ratios for each line and a dynamic ^84^Sr/^86^Sr ratio (Table 1). The latter is obtained by correcting the ^84^Sr/^86^Sr measured in the first line for instrumental mass fractionation using ^86^Sr/^88^Sr measured in the second line as follows:^7,26^
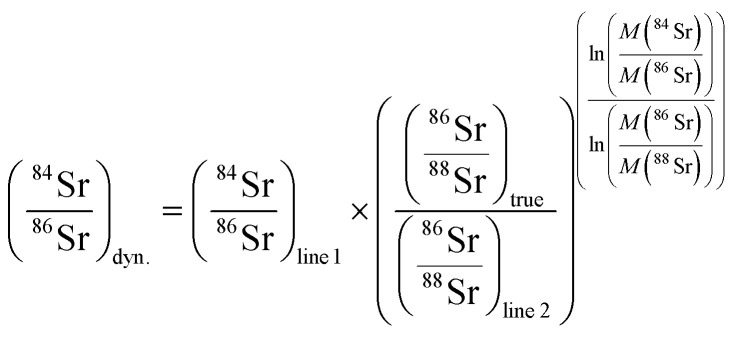


The ^84^Sr/^86^Sr and ^86^Sr/^88^Sr ratios measured in this manner use the same Faraday cups, and so any bias induced by different cup efficiencies cancel out.^7,26^ The ^84^Sr/^86^Sr measurements were performed using 4 s idle time on line 1 and 10 s idle time on line 2, which ensures complete decay of the high ^88^Sr beam measured in the H2 cup, which in line 2 is used to measure the ion beam on the much lower abundance isotope ^86^Sr. Both lines used 4 s integration time and a peak center and lens focus was performed every five blocks. One measurement consists of 25 blocks of 20 cycles each.

The ^87^Sr/^86^Sr measurements used a three-line data acquisition theme following the method described in ref. 7. In this procedure, ^86^Sr is collected in cups H1, C, and L1, respectively, with all other Sr isotopes collected accordingly in each line (Table 1). This procedure allows determining three static ^87^Sr/^86^Sr ratios for each line, as well as a multi-dynamic ^87^Sr/^86^Sr calculated as follows:
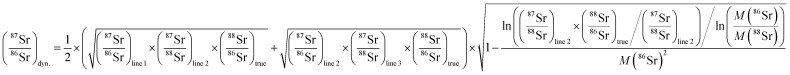
where the subscripts refer to magnet settings of lines 1, 2 or 3, respectively (Table 1). One measurement consists of 25 blocks of 20 cycles with 8 s integration time and 4 s idle time each. Baseline (30 s) measurement, peak center, and lens focus was done every five blocks.

For all Sr isotope measurements, possible isobaric interferences from ^87^Rb on ^87^Sr were monitored using the ion beam intensity on interference-free ^85^Rb. Natural samples might require an Rb-interference correction, however, no ^85^Rb signal was observed in any of the Sr isotope measurements of this study, reflecting the lower ionization potential of Rb, the high purity of the SRM 987 Sr standard and the low Rb blank of our procedure.

### 2.4. Chromium isotope measurements

The Cr isotope measurements were made in static mode following the method described in ref. 18. Approximately 1 μg Cr of NIST SRM 3112a was loaded onto outgassed Re filaments and dried at 0.7 A. The sample was covered with about 3 μl of an Al–Si-gel emitter and dried at 1.2 A for 30 s. Finally, the filament was heated to 2 A for ∼5 s, while preventing the filament from glowing to ensure complete homogenization of sample and emitter, and to maintain stickiness of this mixture on the filament.^18^ For the measurements, filaments were stepwise heated until reaching a signal of 10 V on ^52^Cr using the automatic heat function of the Triton Plus using a slow heating procedure (∼30 min). Ion beams on ^50^Cr, ^52^Cr, ^53^Cr, and ^54^Cr were collected in the L3, C, H1, and H2 cups, respectively, and all cups were connected to 10^11^ Ω feedback resistors. Measurement were typically conducted with ∼10 V on ^52^Cr and correspond signal of 200–250 mV on ^54^Cr. Isobaric interferences from ^50^Ti, ^50^V, and ^54^Fe were monitored using ^49^Ti, ^51^V, and ^56^Fe in cups L4, L1, and H4, respectively. Each measurement consisted of 50 blocks of 30 cycles each with 4 s integration time per cycle for setup #1 and was later reduced to 25 blocks of 30 cycles for setup #2. Baseline measurements (30 s) and a lens focus were performed every three blocks.

For multi-static Cr isotope measurements, we used a 4-line routine outlined in Table 2,^27^ where peak shapes and alignments were adjusted using the focus and dispersion lens system of the Triton Plus before each measurement session (*i.e.*, one sample wheel), similar to multi-dynamic Sr isotope measurements. One measurement consists of 10 blocks with 20 cycles with 8 s integration time and 4 s idle time each. A peak-center and baseline (30 s) measurement was performed every two blocks and a filament and lens focus after five blocks. The final ^53^Cr/^52^Cr and ^54^Cr/^52^Cr ratios are calculated as averages of lines 1–4 for ^53^Cr/^52^Cr and lines 1–3 for ^54^Cr/^52^Cr (Table 2). This difference reflects that measurement of ^54^Cr in a fourth line was not possible due to the instrument's cup setup.

**Table 2:** Cup settings for Cr-isotopic measurements

Line	L3	L2	L1	Center	H1	H2	H3	H4
**Static Cr measurements**								
	^49^Ti	^50^Cr	^51^V	^52^Cr	^53^Cr	^54^Cr		^56^Fe

**Multistatic Cr measurements**								
1	^50^Cr	^51^V	^52^Cr	^53^Cr	^54^Cr		^56^Fe	
2		^50^Cr	^51^V	^52^Cr	^53^Cr	^54^Cr		
3			^50^Cr	^51^V	^52^Cr	^53^Cr	^54^Cr	
4				^50^Cr	^51^V	^52^Cr	^53^Cr	

Final mass fractionation-corrected ratios of ^53^Cr/^52^Cr and ^54^Cr/^52^Cr for all Cr measurements were obtained by internally normalizing to a fixed ^50^Cr/^52^Cr ratio of 0.051859 using the exponential law.^25,28^ During measurements using setup #1, a total of 108 standard measurements were made over a period of 12 months, while with setup #2 65 standard measurements were made during the first three months after installation of the new Faraday cups.

## 3. Results and discussion

### 3.1. Effects of cup deterioration on Sr isotope measurements

**Fig. 1:**
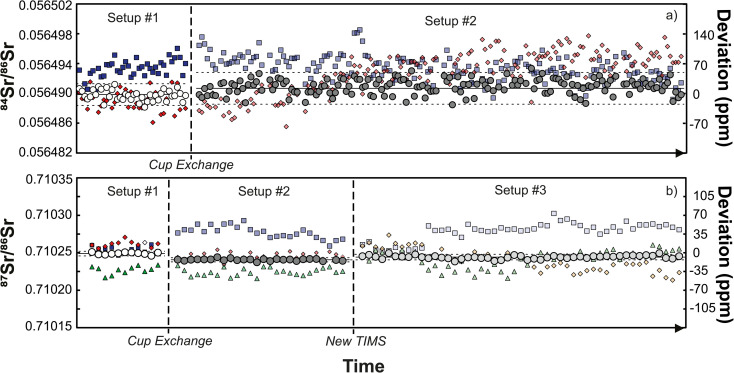
Mass fractionation-corrected ^84^Sr/^86^Sr (a) and ^87^Sr/^86^Sr ratios (b) for the SRM 987 Sr standard for setups #1, #2, and #3. For each setup, results for static measurements from individual lines and the results of the corresponding dynamic measurements are shown. Squares denote static results for line 1, diamonds for line 2, and triangles for line 3. Dashed lines show the external reproducibility (2s.d.) of the dynamic measurements.

The results of static and dynamic ^84^Sr/^86^Sr and ^87^Sr/^86^Sr measurements using two or three instrument setups, respectively, are shown in Fig. 1. While there is significant scatter for the static ^84^Sr/^86^Sr and ^87^Sr/^86^Sr measurements for each setup, the dynamic ^84^Sr/^86^Sr and ^87^Sr/^86^Sr measurements show excellent long-term reproducibility during the 56 months course of this study (for Sr measurements) with mean values of ^84^Sr/^86^Sr = 0.056491 ± 0.000002 (2s.d.; *n* = 203) and ^87^Sr/^86^Sr = 0.710245 ± 0.000004 (2s.d.; *n* = 50). By contrast, static measurements for one particular cup configuration deviate from the dynamic ^84^Sr/^86^Sr and ^87^Sr/^86^Sr ratios by ∼150 and ∼100 ppm, respectively, and also show more scatter, which is predominantly caused by drift of the measured isotope ratios with time. This is particularly evident for the static ^87^Sr/^86^Sr measurements using setups #2 and #3 (Fig. 1). Notably, comparison of the static ^84^Sr/^86^Sr and ^87^Sr/^86^Sr measurements using the three different setups show no obvious difference, *i.e.*, using new Faraday cups in setups #2 and #3 do not appear to have resulted in significant improvement over the measurements performed using setup #1, which used aged Faraday cups. Thus, it is clear that dynamic ^84^Sr/^86^Sr and ^87^Sr/^86^Sr measurements provide much more reproducible and long-term stable results than static measurements (*e.g.*, ref. 29). However, from these measurements alone it remains unclear to what extent the offset and poorer reproducibility of the static measurements is related to deterioration of the Faraday cups.

**Fig. 2:**
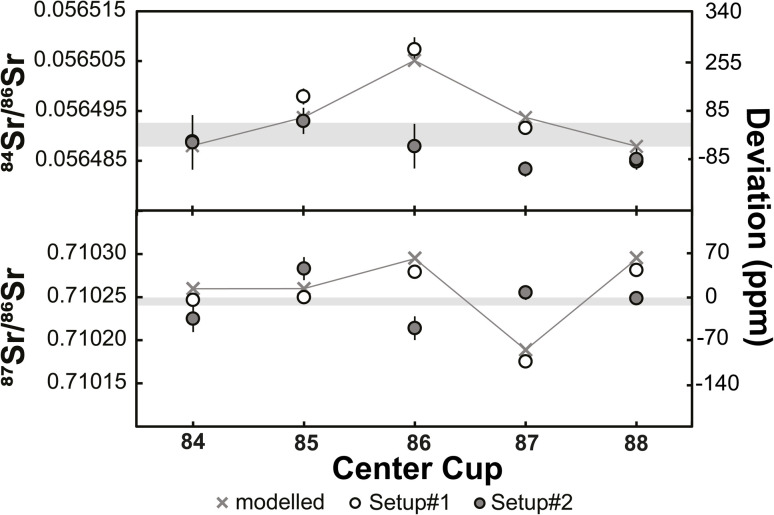
Mass fractionation-corrected ^84^Sr/^86^Sr and ^87^Sr/^86^Sr ratios for NIST SRM 987 measured with different magnet settings (Table 1) and using Faraday cups with extensive pre-usage (setup #1) and new cups (setup #2). Also shown are expected variations if the cup efficiency factor of the center cup is set to 0.9999, while the factor for all other cups is 1. These expected variations are in good agreement with the measured isotope ratios using setup #1. Horizontal grey bands show dynamic results for ^84^Sr/^86^Sr and ^87^Sr/^86^Sr with their respective external reproducibility (±2s.d.).

To assess this effect more rigorously, we performed static ^84^Sr/^86^Sr and ^87^Sr/^86^Sr measurements using setups #1 and #2, and using five different magnetic settings with masses 84 through 88 collected in the center cup (see Table 1). As shown in Fig. 2, for both ^84^Sr/^86^Sr and ^87^Sr/^86^Sr the deviations from the dynamic values are smaller using setup #2 (new cups) compared to setup #1 (old cups), although some deviations remain. To assess whether these deviations can be linked to a specific Faraday cup, we modeled the effect of different Faraday cup efficiency factors on the measured isotope ratios. The cup efficiency can be understood as the ratio of the ion current in the high-ohmage feedback resistor connected to each cup to the ion current that is injected into the cup.^1^ The cup efficiency is not to be confused with the amplifier gain factor, which is a conversion factor between the true input voltage from the cup to the output voltage and is determined before each analytical session (see above). Our calculations show that the different ^84^Sr/^86^Sr and ^87^Sr/^86^Sr ratios measured for the different magnetic settings using setup #1 are well reproduced by modifying the efficiency factor of only the central Faraday cup (Fig. 2). This is consistent with results of a prior study^29^ and the observation that for measurements with either ^84^Sr or ^85^Rb collected in the center cup, the variations among the static and dynamic ^87^Sr/^86^Sr measurements are the smallest (Fig. 2). This is because for these magnetic settings, the center cup is not used for either ^86^Sr, ^87^Sr, or ^88^Sr, which are the three isotopes used for the calculation of mass-fractionation-corrected ^87^Sr/^86^Sr ratios. In detail, the variable ^84^Sr/^86^Sr and ^87^Sr/^86^Sr ratios measured using the different magnetic settings are reproduced by arbitrarily setting the cup efficiency of the center cup to 0.9999, while keeping the cup efficiencies of all other cups at 1. This does not mean that only the center cup is deteriorated, but it shows this cup appears to be affected the most in this specific case. As discussed by ref. 1, the main cause of changes in cup efficiency is the number of ions already collected in a specific cup. This is consistent with the quite extensive usage history of about six years for this set of Faraday cups and with the fact that for most measurements conducted on this instrument, the center cup usually received the highest ion beams. For instance, the instrument has been used for extensive static Cr isotope measurements (see Section 3.2 below), where the most abundant isotope ^52^Cr is collected in the center cup.

We conducted the same measurements with setup #2, *i.e.*, using a new set of Faraday cups. The absolute ^84^Sr/^86^Sr and ^87^Sr/^86^Sr ratios measured deviate from those obtained using setup #1, but as noted above, also deviate from the dynamic results obtained using both setups. However, the deviations observed for setup #2 cannot be attributed to the relative difference in the efficiency of an individual cup. This is not surprising because, given that a new set of Faraday cups has been used, there is no single cup that should be systematically different from all other cups. As such, the differences among the static ^84^Sr/^86^Sr and ^87^Sr/^86^Sr ratios measured for different magnetic settings, and the deviations of these values from the dynamic measurements, most likely reflect small inherent variations in Faraday cup efficiencies related to differences in material and surface properties among the individual cups.

**Fig. 3:**
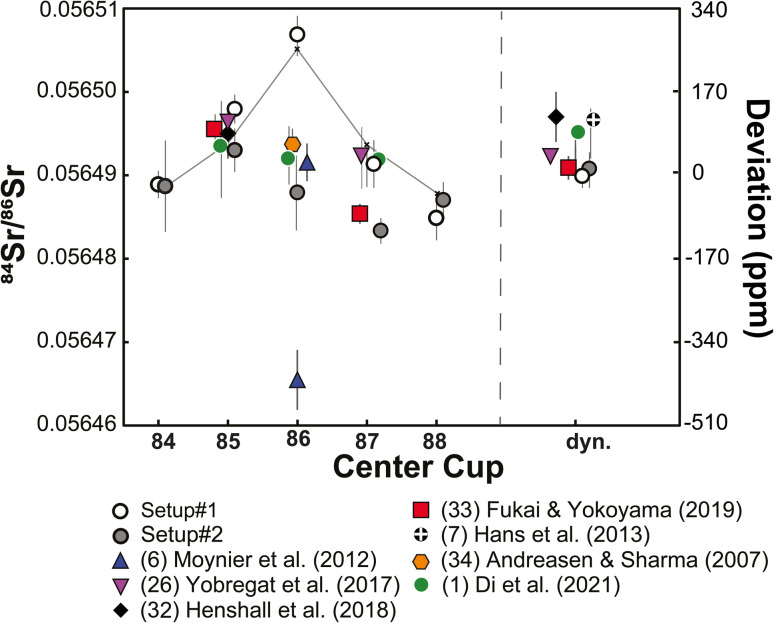
Comparison of ^84^Sr/^86^Sr ratios for NIST SRM 987 measured in this and in prior studies. Note the good agreement of the results except for instrument setups where ^86^Sr is collected in the center cup. Small crosses and solid line are the modelled results from Fig. 2. For details see text.

Fig. 3 compares the ^84^Sr/^86^Sr ratios with different magnet settings of this study to available data from previous studies. Two observations stand out. First, the variable ^84^Sr/^86^Sr ratios reported in prior studies show a similar pattern as our results for different magnetic settings, where the largest offsets are observed when ^86^Sr is measured in the center cup. Of note, when ^85^Rb or ^87^Sr are collected in the center cup, the offsets are much smaller, most likely because the center cup is not used for measurement of ^84^Sr, ^86^Sr, or ^88^Sr, *i.e.*, the three isotopes used for calculation of mass-fractionated ^84^Sr/^86^Sr ratios. Thus, systematic differences for different magnet settings and larger cup deterioration for the center cup are not a feature specific to a single TIMS instrument, but also appear to be present for other TIMS instruments. Second, the dynamic ^84^Sr/^86^Sr measurements of all studies are in very good agreement, suggesting that differences in reported ^84^Sr/^86^Sr ratios predominantly are due to unaccounted variations in Faraday cup efficiencies.

### 3.2. Effect of cup deterioration on Cr isotope measurements

**Fig. 4:**
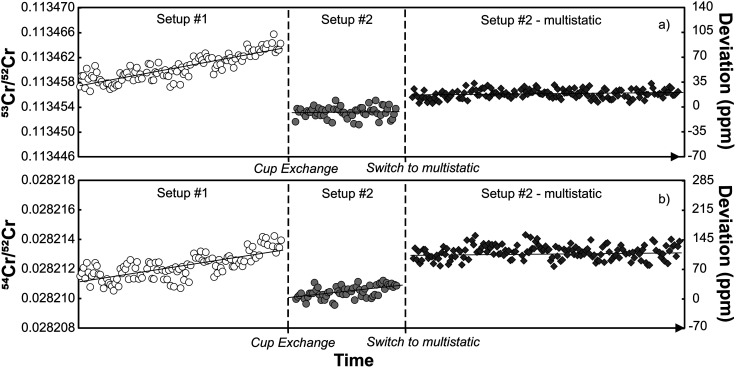
Mass fractionation-corrected ^53^Cr/^52^Cr (a) and ^54^Cr/^52^Cr ratios (b) for the SRM 3112a Cr standard obtained by static measurements using setup #1 and setup #2, and multistatic measurements using setup #2.

**Fig. 5:**
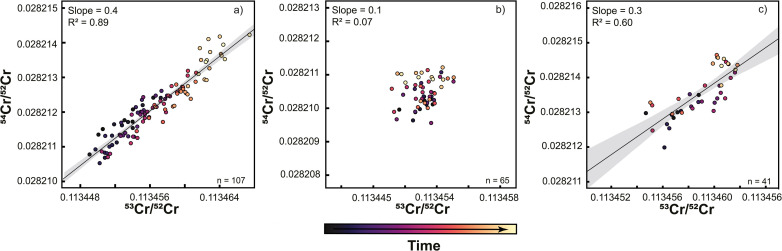
Mass fractionation-corrected ^53^Cr/^52^Cr and ^54^Cr/^52^Cr ratios of NIST SRM 3112a obtained by static measurements using setup #1 (a) and setup #2 (b and c). Note the strong correlation of ^53^Cr/^52^Cr and ^54^Cr/^52^Cr obtained using setup #1 (a), which disappears for the initial measurements using setup #2 (two months) (b), but reappears relatively quickly after about eight months (c). Color-coding of data points indicates the relative time of individual measurements for each setup, demonstrating that the correlation of ^53^Cr/^52^Cr and ^54^Cr/^52^Cr ratios reflects a temporal drift of both ratios to higher values over time.

During the course of the Sr isotope measurements described above, the TIMS instrument in Münster (setups #1 and #2) was also extensively used for Cr isotope measurements. Given the evidence for deterioration of the central cup based on the Sr isotope measurements, the question arises how this deterioration affected the Cr isotope measurements. As shown in Fig. 4, both the ^53^Cr/^52^Cr and ^54^Cr/^52^Cr measured for the SRM 3112a standard using setup #1 drift over time to higher values, resulting in a strong positive correlation between these two ratios (Fig. 4 and 5). This correlation has also been observed in several prior studies.^16,27,30^ Immediately after the cup exchange (setup #2), the measured ^53^Cr/^52^Cr and ^54^Cr/^52^Cr ratios drop substantially by ∼90 ppm and ∼130 ppm, respectively, to values that are even lower than the value at the beginning of the measurement series using setup #1. However, even for setup #2 a slight drift in measured ^54^Cr/^52^Cr ratios can be observed, and after about 8 months of use, a weak correlation between the measured ^53^Cr/^52^Cr and ^54^Cr/^52^Cr ratios emerges again (Fig. 5). As shown in Fig. 5, this drift and the resulting correlation is a temporal effect, as later measurements systematically yielded higher ^53^Cr/^52^Cr and ^54^Cr/^52^Cr ratios compared to the earlier measurements.

**Fig. 6:**
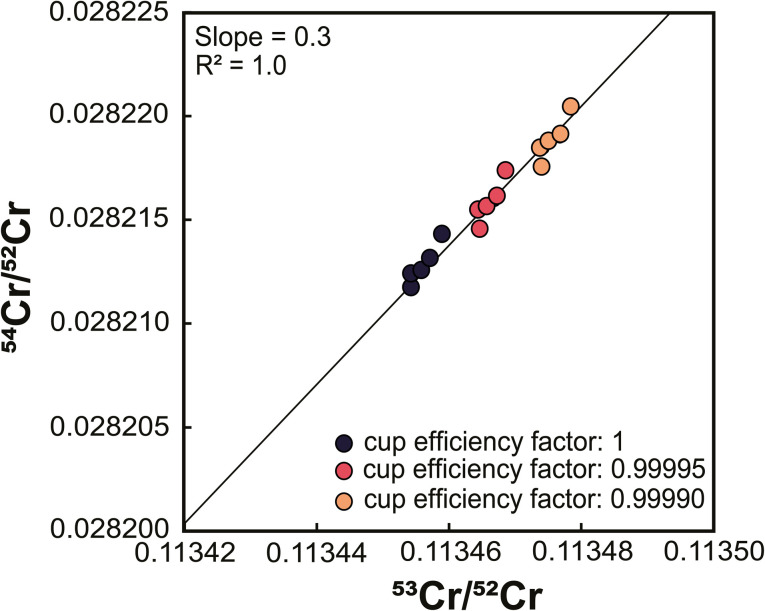
Effect of changing Faraday cup efficiency on mass fractionation-corrected ^53^Cr/^52^Cr and ^54^Cr/^52^Cr ratios. Calculations assume that the cup efficiency of only the center cup changes and show that factors of 0.9999 and 0.99995, instead of 1, result in a positive correlation between ^53^Cr/^52^Cr and ^54^Cr/^52^Cr ratios similar to the correlation observed for the standard measurements.

To assess whether the correlation of ^53^Cr/^52^Cr *versus*^54^Cr/^52^Cr ratios is due to Faraday cup deterioration, we calculated the change of both ratios resulting from a different cup efficiency of the central cup. As shown above, for the Sr isotope measurements using setup #1 we found that a cup efficiency of 0.9999 for the center cup, and 1 for all other cups, can account for the observed variations in measured ^84^Sr/^86^Sr and ^87^Sr/^86^Sr ratios. We find that these cup efficiencies result in higher measured ^53^Cr/^52^Cr and ^54^Cr/^52^Cr ratios compared to the ratios obtained with an efficiency of 1 for all Faraday cups, and a positive correlation between the two ratios with a slope that is in good agreement with the slopes observed for measurements with setup #1 and the later measurements with setup #2 (Fig. 6). Thus, as for Sr isotopes, the observed offsets in measured Cr isotope ratios can be attributed to Faraday cup deterioration of mainly the center cup.

The correlation of ^53^Cr/^52^Cr and ^54^Cr/^52^Cr ratios has most commonly been attributed to residual mass fractionation that has not been accounted for by the correction for instrumental mass fractionation.^16,27,30^ However, our results show that at least for static measurements this correlation more likely results from changes in the Faraday cup efficiency of especially the center cup, which is used to collect the ion beam of the most abundant Cr isotope, ^52^Cr, and is, as such, most susceptible to cup deterioration.

**Fig. 7:**
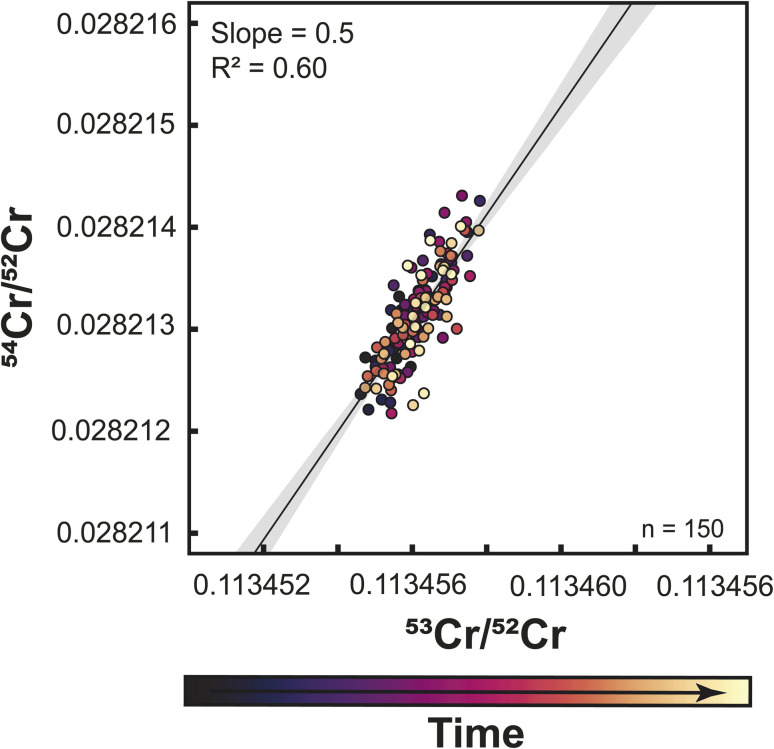
Multistatic mass-fractionation-corrected ^53^Cr/^52^Cr and ^54^Cr/^52^Cr ratios measured using setup #2. As for the static measurements, there is a residual correlation between ^53^Cr/^52^Cr and ^54^Cr/^52^Cr, but the overall variations are smaller and the correlation does not reflect a temporal change in measured ratios, suggesting this correlation is due to residual mass fractionation effects.

To overcome these problems inherent to static Cr isotope measurements, several studies used a multi-static setup, where Cr isotopes are measured in four or five different lines.^27,31^ The same amount of sample (1 μg) was loaded onto one filament for multistatic measurements as for single static measurements, but the measurement time is four times as long as one static measurement. The idea of this approach is that the effects of different cup efficiencies should average out, as through the multi-line data acquisition all Cr isotopes are on average measured using the same set of Faraday cups. To assess if this is the case, we performed multi-static Cr isotope measurements using setup #2, which were all conducted after the static measurements conducted with this setup. We find that over a period of 9 months, both the ^53^Cr/^52^Cr and ^54^Cr/^52^Cr ratios no longer exhibit drift, thus allowing precise measurements of both ratios. However, when the multi-static ^53^Cr/^52^Cr and ^54^Cr/^52^Cr ratios are plotted against each other, a correlation with a similar slope as before is still apparent (Fig. 7). A similar observation has been made by ref. 27, using a similar setup as in this study. Importantly though, unlike for the static measurements, for the multi-static measurements the ^53^Cr/^52^Cr and ^54^Cr/^52^Cr ratios do not evolve over time and, as such, are unlikely to be related to the increasing aging of the Faraday cups. This is consistent with the idea that the effects of Faraday cup efficiencies should nearly cancel out in a multi-static measurement setup and suggests that the observed residual ^53^Cr/^52^Cr *versus*^54^Cr/^52^Cr correlation in part reflects unaccounted mass fractionation effects.^27^ Nevertheless, in the dataset of this study the overall range of ^53^Cr/^52^Cr and ^54^Cr/^52^Cr ratios is larger for the static than for the multi-static measurements, indicating that at least for single-line static measurements, aging of the Faraday cups is the dominant factor causing the observed ^53^Cr/^52^Cr *versus*^54^Cr/^52^Cr correlation.

## 4. Conclusions

This study compares the results of long-term high-precision Sr (*i.e.*, ^84^Sr/^86^Sr, ^87^Sr/^86^Sr) and Cr (*i.e.*, ^53^Cr/^52^Cr, ^54^Cr/^52^Cr) isotope measurements for the NIST SRM 987 and NIST SRM 3112a standards, respectively, acquired on two thermal ionization mass spectrometers equipped with three different sets of Faraday cups having varying usage histories of between six years and one month. This extensive data set allows assessing the influence of Faraday cup deterioration on the precision and accuracy of the isotope measurements. Our results show that for static measurements drift and scatter of mass fractionation-corrected ^84^Sr/^86^Sr and ^87^Sr/^86^Sr as well as ^53^Cr/^52^Cr and ^54^Cr/^52^Cr ratios can be attributed to increasing deterioration of predominantly the center cup. For Sr isotopes, this problem can be overcome by dynamic measurements, which in this study provided stable and reproducible results over a period of 56 months for all three sets of Faraday cups used. For Cr isotopes, we find that the correlation between mass fractionated-corrected ^53^Cr/^52^Cr and ^54^Cr/^52^Cr ratios observed for static measurements in several prior studies can also largely be attributed to deterioration of the center cup. This residual correlation disappears when new Faraday cups are used, but appears again after only a few months of use and increases over time. Multistatic measurements minimize the long-term drift in mass fractionation-corrected ^53^Cr/^52^Cr and ^54^Cr/^52^Cr ratios, but a small residual correlation between ^53^Cr/^52^Cr and ^54^Cr/^52^Cr remains, which most likely reflects residual mass fractionation. Finally, the comparisons to literature data for both Sr and Cr isotopes reveal that the effects of Faraday cup deterioration described here are not specific to the TIMS instruments of this study, but appear to be relatively common among TIMS instruments used for high-precision isotope measurements in other laboratories.

## Author contributions

Jonas M. Schneider: conceptualization, formal analysis, investigation, methodology, visualization, writing – original draft. Thorsten Kleine: conceptualization, funding acquisition, project administration, resources, supervision, writing – review & editing.

## Conflicts of interest

The authors declare that they have no known competing financial interests or personal relationships that could have appeared to influence the work reported in this paper.
